# ACOT7, a candidate and novel serum biomarker of Alzheimer’s disease

**DOI:** 10.3389/fnagi.2024.1345668

**Published:** 2024-07-04

**Authors:** Jintao Wang, Yong Feng, Yingni Sun

**Affiliations:** ^1^Department of Pharmacy, First People’s Hospital of Wenling, Wenling, China; ^2^Department of Medical Research, Qingdao Huangdao People’s Hospital, Qingdao, China; ^3^State Key Laboratory of Chemical Resources Engineering, Beijing University of Chemical Technology, Beijing, China; ^4^Beijing Handian Pharmaceutical Co, Ltd., Beijing, China; ^5^School of Life Sciences, Ludong University, Yantai, China

**Keywords:** Alzheimer’s disease, ACOT7, biomarker, diagnosis, serum

## Abstract

Alzheimer’s disease (AD) is the most common fatal neurodegenerative disease among the elderly worldwide, characterized by memory and cognitive impairment. The identification of biomarkers for AD is crucial and urgent to facilitate the diagnosis and intervention. The aim of this study was to evaluate the diagnostic value of acyl-Coenzyme A thioesterase 7 (ACOT7) as a serum biomarker for the prediction of AD. In our study, we observed a significant increase in ACOT7 expression in patients (*n* = 366) with AD and animal (*n* = 8–12) models of AD, compared to the control group. A significant negative correlation was found between ACOT7 levels and Mini-Mental State Examination (MMSE) scores (*r* = −0.85; *p* < 0.001). The analysis of the receiver operating characteristic curve (ROC) showed that the area under the curve (AUC) for ACOT7 was 0.83 (95% confidence intervals: 0.80–0.86). The optimal cut-off point of 62.5 pg./mL was selected with the highest sum of sensitivity and specificity. The diagnostic accuracy of serum ACOT7 for AD was 77% (95% confidence intervals: 72–82%), with a sensitivity of 80% (95% confidence intervals: 75–84%) and a specificity of 74% (95% confidence intervals: 69–79%). Moreover, the ROC analysis showed that the AUC of Aβ_42/40_ ratio is 0.70, and the diagnostic accuracy was 72%, with 69% sensitivity and 76% specificity. Compared with the AD traditional marker Aβ_42/40_ ratio, ACOT7 shows better superiority as a new serum candidate biomarker of AD. By suppressing the ACOT7 gene, our study provides evidence of the involvement of ACOT7 in the metabolism of amyloid precursor protein (APP), resulting in alterations in the expression levels of Aβ_42_, BACE1 and βCTF. ACOT7 has the ability to modulate the amyloidogenic pathway of APP metabolism, while it does not have an impact on the non-amyloidogenic pathway. In conclusion, the findings of our study suggest that serum ACOT7 may serve as a promising and non-invasive biomarker for AD.

## 1 Introduction

The incidence of Alzheimer’s disease (AD) is steadily increasing in line with the aging population, and it is projected that there will be approximately 131.5 million AD patients worldwide by 2050. This not only significantly impacts the physical and psychological well-being of older individuals but also imposes substantial medical and financial burdens on both society and affected families (Bondi et al., 2017; Scheltens et al., 2021). Despite extensive research, the intricate pathogenesis of AD remains elusive, although the amyloid β (Aβ) hypothesis stands as the most widely accepted theory (Reiss et al., 2018; Zhang and Zheng, 2019). Numerous drug development efforts have been undertaken to combat AD; however, none have successfully prevented or reversed disease progression thus far (Lyketsos, 2020; Srivastava et al., 2021; Yu et al., 2021). Clinical diagnosis typically occurs during the middle to late stages of AD when existing treatments struggle to achieve satisfactory efficacy. Consequently, early detection and intervention hold promise for effective AD management.

Currently, the NINCDS ADRDA standard is widely used in the clinical diagnosis of AD (Dubois et al., 2007), relying on the manifestation of symptoms observed during clinical assessment. In order to eliminate other conditions that may contribute to dementia, a comprehensive approach is taken, including clinical physical examination, auxiliary clinical examination, and neuropsychological testing. However, the pathological alterations in AD manifest as early as 20–30 years prior to the onset of symptoms. Therefore, it is imperative to identify a methodology that can aid in the diagnosis of AD. And there is a growing focus on searching for a simple and non-invasive peripheral blood biomarker that accurately reflects the pathological process of AD.

The main function of acyl-Coenzyme A thioesterases (ACOTs) involved in the metabolism of long-chain fatty acids is to catalyze the hydrolysis of acyl-coenzyme A thioesterases into corresponding free fatty acids (FFA) and coenzyme A (CoA), thereby regulating the levels of FFA and CoA in cells (Kuramochi et al., 2002; Hunt et al., 2005). ACOT7, a member of the ACOTs family II, is highly expressed specifically in the brain, which is the main source of ACOTs activity in the brain (Hunt et al., 2007). ACOT7 had high activity against middle and long chain (C8-C18) acyl CoA (Yamada et al., 1999). Previous studies have confirmed that ACOT7 is highly expressed in neurons and oligodendrocytes, and can protect nerve cells from the toxicity of the accumulation of long chain fatty acids (Hein et al., 2008; Ellis et al., 2013). It has also been shown that ACOT7 plays an important role in myelin development and maintenance (Shi et al., 2018). In addition, the cytoplasmic activity of ACOT7 is regulated by the energy molecule ATP, which is consistent with the high sensitivity of myelination to abnormal energy metabolism (Nave and Werner, 2021). A recent study showed that ACOT7 formed fibrils in the presence of its substrate arachidonoyl-CoA, which probably act as functional amyloid (Kumar et al., 2023). Given that, we speculated whether ACOT7 could act as a valuable biomarker in AD. Thus, we detect the levels of ACOT7 from serum samples in AD patients and healthy controls, as well as in animal and cell models, and we also evaluated the diagnostic values of ACOT7 as an AD biomarker through the receiver operator characteristic curve (ROC) analysis. The correlation analysis between ACOT7 levels and cognitive decline was also performed to determine whether higher ACOT7 expression was associated with more severe disease or not. It was very meaningful for the discovery of ideal serum biomarkers of AD.

There exist two primary pathways for the metabolism of APP in living organisms, namely the non-amyloidogenic pathway and the amyloidogenic pathway (Zhang and Song, 2013; Chau et al., 2023). In the non-amyloidogenic pathway, APP is cleaved by α-secretase and γ-secretase, leading to the suppression of Aβ production. In the amyloidogenic pathway, APP is cleaved by β-secretase and γ-secretase to generate Aβ. In a normal physiological state, the metabolism of APP is predominantly regulated by the non-amyloidogenic pathway. In pathological states, there is a notable upregulation of the amyloidogenic pathway of APP, resulting in the generation of a large amount of Aβ, and thus forming Aβ oligomers and Aβ plaque deposits. Aberrant metabolism of APP may result in the accumulation of Aβ peptides in both the cerebrospinal fluid (CSF) and blood. In the present study, we conducted a preliminary and exploratory study on the association between ACOT7 and Aβ production, which paved the way for subsequent comprehensive analysis and study of the specific molecular mechanism of ACOT7 in the occurrence and development of AD.

## 2 Materials and methods

### 2.1 Entry criteria

The participants were categorized into two groups, namely “AD” (Alzheimer’s disease) and “Control (cognitively normal),” based on their cognitive impairment level. Prior to the neuropsychological examination, these participants completed the informed consent, submitted their medical history, and underwent a thorough neurological physical examination.

All the participants were required to be in a stable physical condition, without any recent acute medical events, and without any notable acute fluctuations in cognitive functioning. The diagnosis was based on some clinical diagnostic criteria such as NINCDS-ADRDA and DSM-IV-TR. In the later follow-up, diagnostic support was also confirmed by cerebrospinal fluid examination, structural neuroimaging MRI and neuro-molecular imaging PET, as well as some other relevant tests. The follow-up was performed once a month after first examination in all participants. All participants underwent cognitive function screening using the Mini-Mental State Examination (MMSE) test, regardless of self-reported or clinically observed cognitive impairment. If patients exhibited cognitive impairment with a MMSE score above 16 (including 16) (Creavin et al., 2016), they underwent a thorough neuropsychological assessment to determine the severity of their cognitive impairment, including MMSE, Alzheimer’s Disease Assessment Scale (ADAS), Abbreviated Barcelona Test (ABT), Global Dementia Staging (GDS), Functional Assessment Staging (FAST), Clinical Dementia Rating (CDR), Rapid Disability Rating Scale-2 (RDRS-2), Blessed Dementia Rating Scale (BDRS), Interview for Deterioration in Daily life in Dementia (IDDD), Geriatric Evaluation by Relatives Rating Instrument (GERRI), Geriatric Depression Scale (GDS), and Zung Self-Rating Anxiety Scale (ZSRAS). The threshold of16 was mainly determined by the consensus of clinicians according to the international common diagnostic criteria combined with Chinese national conditions. Cognitively normal individuals were assigned to the “cognitively normal” group, which served as the control group (MMSE = 27–30).

All patients with confirmed cognitive impairment underwent routine laboratory screening, including blood tests (CBC, liver function, kidney function, lipids, blood glucose, thyroid function, HIV, syphilis, hepatitis C/B) and electrocardiogram. If the patient exhibits abnormalities in any of the above tests, it is crucial to address and rectify these alterations prior to conducting a cognitive assessment. If the indicators cannot be improved, the enrolled subject will be excluded. Both patients and their families gave informed consent by voluntarily signing the consent form. This study is just a small part of a prospective national research project on the pathogenesis of AD, which was approved in 2016. A total of 366 AD subjects and 316 non-demented healthy controls matched for age (≥60), gender, BMI and education were recruited in Shandong local hospitals, and the differences between groups were controlled. Detailed demographic information of the subjects enrolled in the study is presented in Table 1.

**Table 1:** Demographics characteristics of the study samples.

	Control	AD
Number	316	366
Age, years	68.1 ± 6.5	69.8 ± 4.7
Sex, M/F	162/154	176/190
BMI	27.3 ± 5.5	26.5 ± 5.1
MMSE, points	28.5 ± 0.6	18.1 ± 2.9
Education, years	7.0 ± 3.2	6.2 ± 4.9

Data presented as mean ± SD where appropriate. Student’s *t* test; AD, Alzheimer’s disease; M/F, male/female; BMI, body mass index; MMSE, mini-mental status examination.

### 2.2 Collection of blood samples

After the informed consents, serum samples of AD and control group were collected. Specific collection methods were as follows: 3–4 mL venous blood was collected in 5 mL disposable vacuum negative pressure vessel, placed at room temperature for 30 min, centrifuged at 3000 r/min for about 15 min, and the serum was separated on a cleaning workbench, and immediately transferred to −20°C for storage.

### 2.3 Animal models

APP/PS1 double transgenic mice and age-matched wild-type (WT) mice aged 8 months were purchased from the Jackson Laboratory Company, and the study was performed after the mice were allowed to acclimate for 2 weeks. The brain tissues and serum samples were collected. All the experiments were approved according to the institutional guidelines of the Experimental Animal Center of Ludong University.

### 2.4 RNA interference

SK-N-SH and SK-N-SH APPwt cells were grown in DMEM, plus 10% fetal bovine serum (Hyclone, Los Angeles, CA, United States) and 100 U/mL penicillin/streptomycin. For ACOT7 knockdown, specific siRNAs for human ACOT7 and negative control were all obtained from Invitrogen (Carlsbad, CA, USA). Briefly, 10 μL lipofectamine RNAiMAX and 5 μL siACOT7 or siControl with DMEM (no FBS) were diluted in 250 μL DMEM (no FBS), and then were mixed and incubated at the room temperature for 20 min. The mixture with final concentration of siRNA 70 nmol/L and 2 μL lipofectamine RNAiMAX was incubated for 20 min and then added to 80–90% confluent SK-N-SH APPwt cells. Five hours after transfection, the medium was changed to the fresh complete medium for an additional 20, 44, or 68 h.

### 2.5 Western blot analysis

The whole brains were quickly removed after the mice were decapitated, and the hippocampus and cortex were quickly separated on ice. The tissue homogenates from hippocampus were prepared by ultrasonic crushing (80 w, 6 s × 4 times, 30 s interval) by adding precooled protein lysates at 1:8 by weight/volume ratio, and the homogenates from cortex were prepared by motor homogenization (12,000 r/min, 6 s × 4 times, 30 s interval). The supernatant was obtained after the centrifugation (12,000 g × 10 min, 4°C). Protein was quantified by Bradford method and protein concentration was calculated according to the standard curve. In the cell and serum samples, total protein was extracted using RIPA buffer and quantified by the same method.

After the completion of electrical transfer, the PVDF membrane was put into TBST containing 5% skim milk powder and sealed by shaking at room temperature for more than 2 h, and incubated with primary antibody at 4°C overnight and with horseradish peroxidase labeled secondary antibody for 2 h by shaking at room temperature. The PVDF membrane was removed and washed with TBST at room temperature for 5 min × 5 times. The Millipore chemiluminescence solution was uniformly added to the PVDF membrane. Digital images were obtained with the LAS4000 FujiFilm imaging system (FujiFilm, Japan), and the densitometric analysis was made by Quantity-One software (Bio-Rad, United States).

### 2.6 ELISA detection

ACOT7 levels in the serum of 366 patients diagnosed with AD and 316 normal controls were quantified using the enzyme-linked immunosorbent assay (ELISA). The reagents should be transferred and stored at room temperature to achieve equilibrium for a minimum of 30 min. The standards and test samples were pipetted into 96-well plates pre-coated with anti-ACOT7 antibody and incubated for 2 h at 37°C subsequently. After the removal of the liquid from each well, 100 μL of biotin-conjugated antibody specific for ACOT7 was added and incubated for 1 h at 37°C. Then, they were mixed gently until the solution appeared uniform at room temperature, and this was then washed with wash buffer three times. After that, the avidin conjugated HRP was added and incubated for 1 h at 37°C. To remove any unbound avidin-enzyme reagent, the aspiration/wash process was repeated five times. TMB substrate was added and incubated for 15–30 min at 37°C. After that, the stop solution was added. The optical density was determined using an MQX200 microplate reader (Bio-Tek, United States) set to 450 nm. The serum level of ACOT7 in the samples was interpolated from kit-specific standard curves generated using GraphPad Prism software. Similarly, the serum levels of Aβ_42_ and Aβ_40_ were measured. Aβ_42/40_ ratio was calculated.

### 2.7 Statistical analysis

The data were analyzed using SPSS 13.0 software. Comparison between the groups was conducted using Student’s t-test and Mann–Whitney U-test. The correlation analysis was performed with the Spearman correlation coefficient. ROC analysis was used to assess the sensitivity and specificity of ACOT7 in the diagnosis of AD, as well as Aβ_42/40_ ratio. The optimal cut-off value was selected with the highest sum of sensitivity and specificity. *p* < 0.05 was considered statistically significant.

## 3 Results

### 3.1 Demographic characteristics

The educational level and Body Mass Index (BMI) between AD and Control group were similar. See Table 1 for details. All samples were matched for age, gender distribution, BMI and education. The MMSE score is a widely recognized and important measure of cognitive function. The AD group had a significantly lower MMSE score in comparison to the control group.

### 3.2 ACOT7 expression in AD animal models

**Figure 1:**
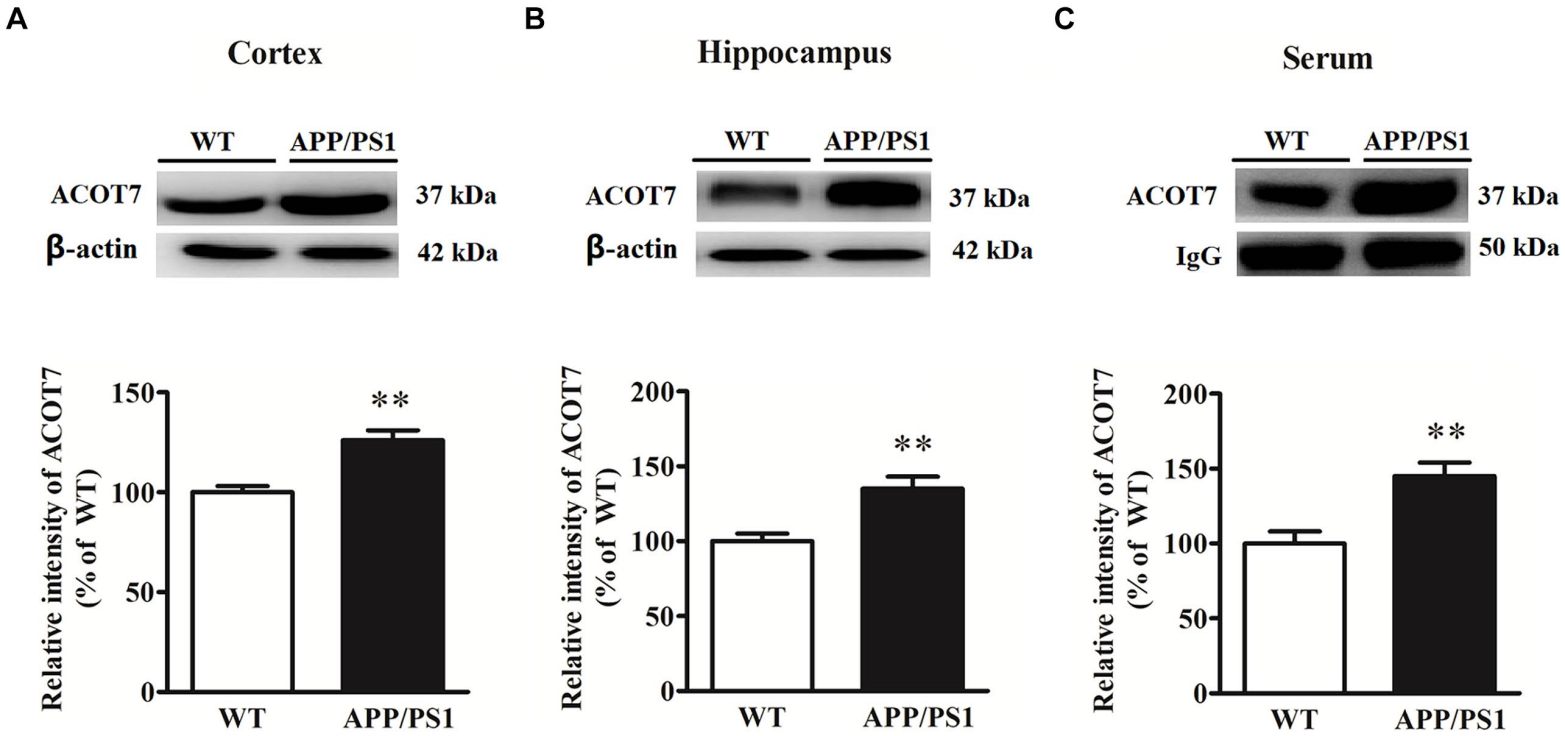
Western blot analysis of ACOT7 in the cortex **(A)**, hippocampus **(B)** and serum **(C)** samples from APP/PS1 transgenic mice and age-matched wild-type (WT) mice. Quantitative results were normalized to β-actin/IgG expression. Values were expressed as percentages compared to the WT group (set to 100%) and represented as means ± SEM. *n* = 8–12 per group. ^**^p < 0.01.

The results showed that the expression of ACOT7 was increased by 26% ± 6% (*p* = 0.0030) in the cortical tissues of APP/PS1 transgenic mice compared to age-matched WT mice, with a significant difference. ACOT7 expression in the hippocampus of APP/PS1 transgenic mice exhibited a notable increase of 35% ± 6% (*p* = 0.0016) in comparison to age-matched WT mice. Similarly, the serum levels of ACOT7 were obviously 45% ± 12% (*p* = 0.0015) higher than those in WT mice. Specific results are shown in Figure 1.

### 3.3 Increased ACOT7 serum levels in AD patients

**Figure 2:**
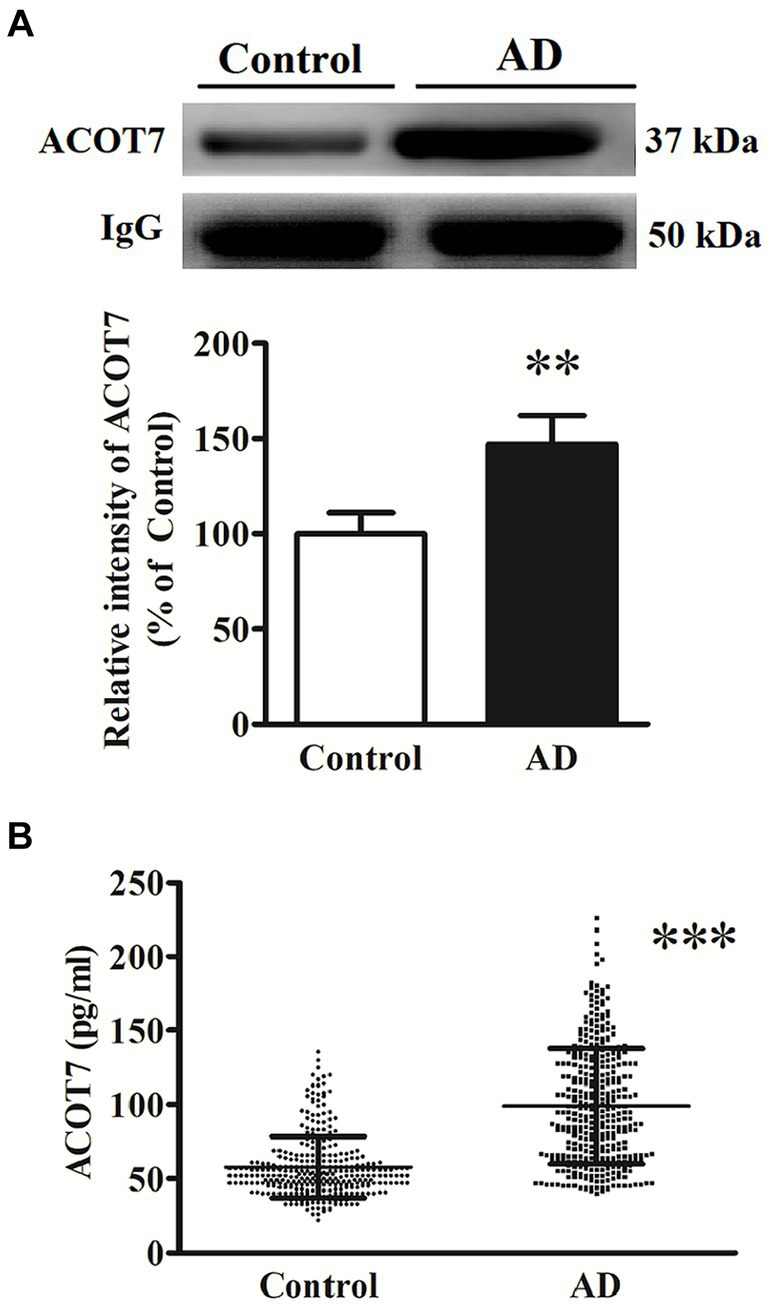
**(A)** Western blot analysis of ACOT7 in the serum samples from AD patients and the controls. Quantitative results were normalized to IgG expression. Values were expressed as percentages compared to the controls (set to 100%) and represented as means ± SEM. *n* = 10–15 per group. ^**^p < 0.01. **(B)** ACOT7 levels in serum were presented as scatter plots for AD patients and healthy controls. ^***^p < 0.001.

The elevated serum level of ACOT7 was subsequently confirmed through western blot and ELISA analyses in both AD patients and control subjects. Western blot analysis revealed a statistically significant increase in serum levels by 47% (*p* = 0.0098) (Figure 2A) in AD patients compared to the controls. Meanwhile, the serum level of ACOT7 was detected by ELISA (Figure 2B), which showed that ACOT7 level was markedly higher in AD than that in the control group (Control: 57.7 ± 20.6 pg./mL, AD: 99.0 ± 39.1 pg./mL, *p* < 0.001).

### 3.4 Correlation analysis

**Figure 3:**
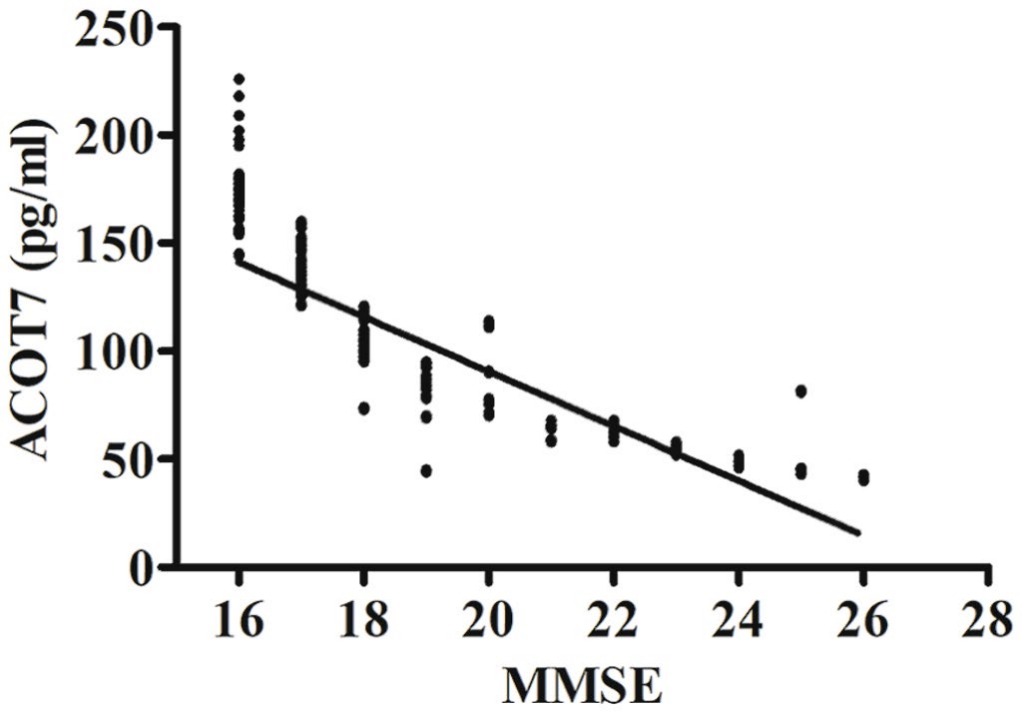
Correlation analysis between ACOT7 serum levels and MMSE scores. Correlation was assessed using the Spearman correlation coefficient. The concentration of ACOT7 in serum was plotted against MMSE score for each patient. A significant negative correlation between serum ACOT7 level and MMSE score (*r* = −0.85, *p* < 0.001) was observed. Correlation lines are also shown.

Correlation analysis showed that serum ACOT7 level was negatively correlated with MMSE scores (r = −0.85, p < 0.001). The differences were statistically significant. The relevant scatter plots are shown in Figure 3.

### 3.5 ROC analysis

**Figure 4:**
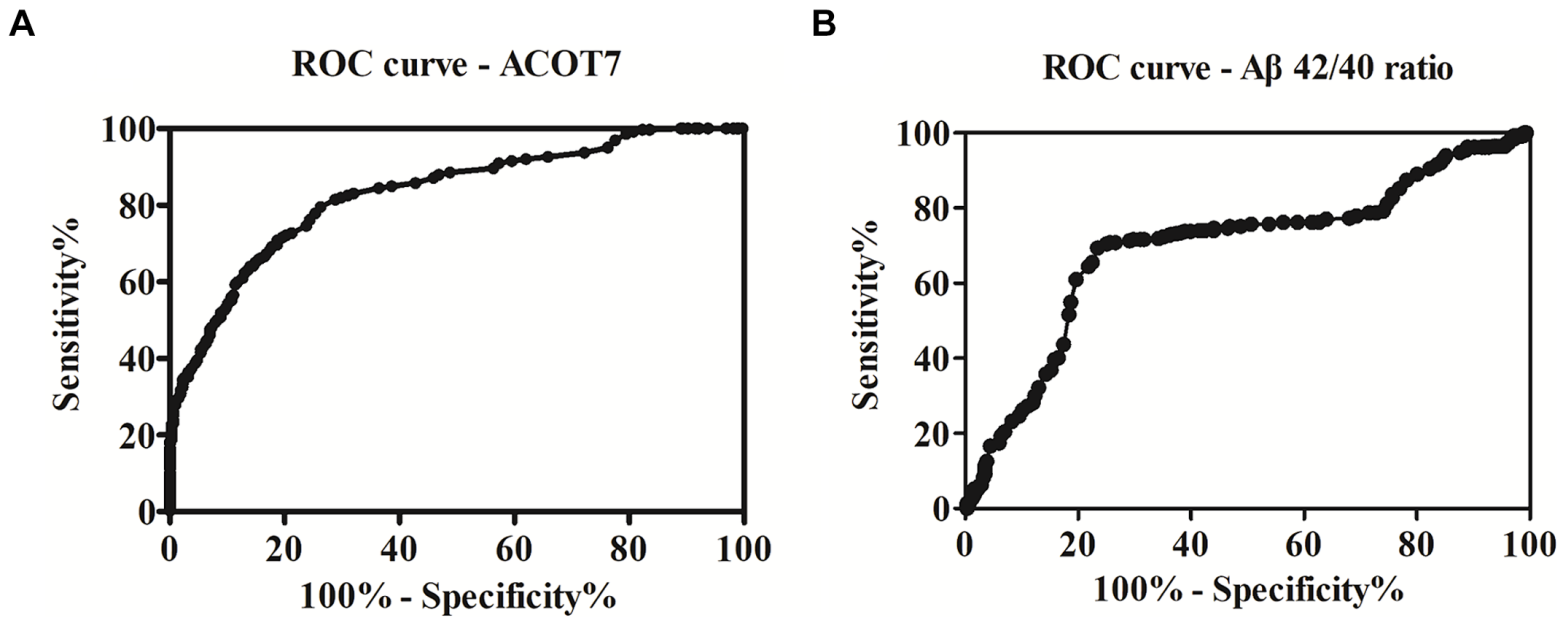
ROC curve analysis and the prediction of the presence of AD. **(A)** The AUC of ACOT7 was 0.83. The optimal cut-off value (62.5 pg./mL) was selected. The diagnostic accuracy for ACOT7 in serum was 77% with the sensitivity and specificity 80 and 74%, respectively. **(B)** The AUC of the traditional marker (Aβ_42/40_ ratio) was 0.70. The diagnostic accuracy for Aβ_42/40_ ratio in serum was 72%, with 69% sensitivity and 76% specificity.

To assess the potential diagnostic utility of ACOT7 as a biomarker for AD, the ROC curve (Figure 4A) was constructed based on the ELISA results. The area under the curve (AUC) was calculated to evaluate the diagnostic performance. ROC curve analysis showed that the AUC of ACOT7 was 0.83 (95% confidence intervals: 0.80–0.86), and the optimal cut-off point with the highest sum of sensitivity and specificity was 62.5 pg./mL. Meanwhile, the diagnostic accuracy of serum ACOT7 in distinguishing AD from controls was found to be 77% (95% confidence intervals: 72–82%), with 80% (95% confidence intervals: 75–84%) sensitivity and 74% (95% confidence intervals: 69–79%) specificity, respectively. In addition, ROC curve analysis of AD traditional marker (Aβ_42/40_ ratio) was also made (Figure 4B). Our study showed that the AUC of Aβ_42/40_ ratio was 0.70, and the diagnostic accuracy was 72%, with 69% sensitivity and 76% specificity. In contrast, ACOT7 displayed the better superiority as an AD serum biomarker.

### 3.6 ACOT7 promoted Aβ metabolism

**Figure 5:**
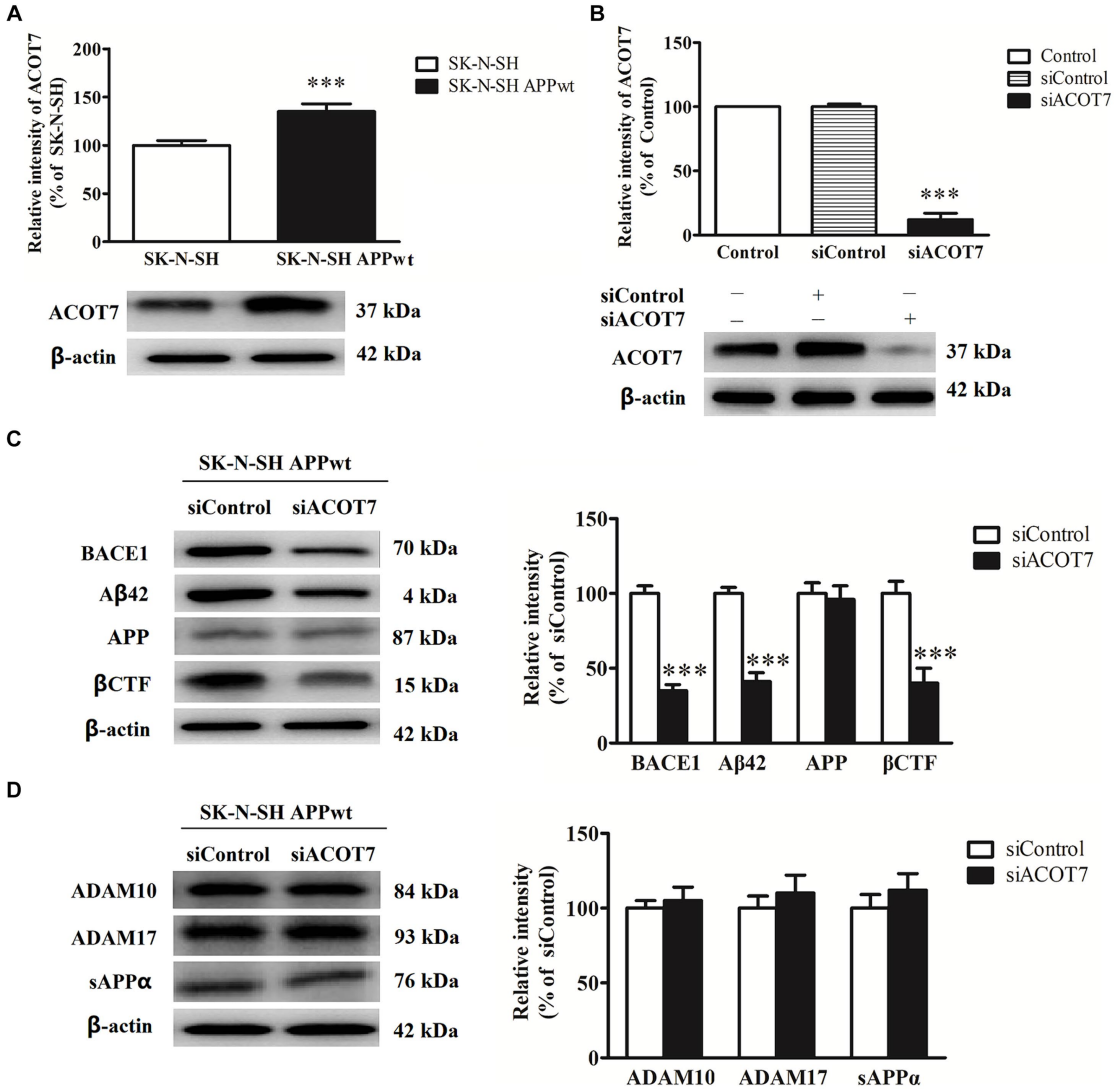
ACOT7 affects the amyloidogenic pathways of APP metabolism. **(A)** Representative Western blots and quantitative results of ACOT7 in SK-N-SH cells and SK-N-SH APPwt cells. **(B)** Representative Western blots and quantitative results of ACOT7 after SK-N-SH APPwt transfected with siControl and siACOT7. **(C)** Representative Western blots and quantitative results of BACE1, Aβ_42,_ βCTF and APP after SK-N-SH APPwt cells were transfected with siControl or siACOT7. **(D)** Representative Western blots and quantitative results of ADAM10, ADAM17 and sAPPα after SK-N-SH APPwt cells were transfected with siControl or siACOT7. ^***^p < 0.001.

In the present study, RNA interference was employed to investigate the impact of ACOT7 knockdown on APP metabolism in neuroblastoma SK-N-SH APPwt cells. The level of ACOT7 expression exhibited a 35 (*p* = 0.0040) increase in SK-N-SH APPwt cells when compared to SK-N-SH cells (Figure 5A). Conversely, when ACOT7 was knocked down with siACOT7, the expression of ACOT7 decreased by 88% (*p* < 0.0001) in SK-N-SH APPwt cells compared to the cells transfected with siControl (Figure 5B). The impact of silencing ACOT7 on the levels of β-secretase (BACE1), Aβ_42_, APP and βCTF was also investigated. The results indicated a significant decrease (p < 0.0001) in BACE1, Aβ_42_ and βCTF levels in the siACOT7 group when compared to the siControl group (Figure 5C), and no significant changes in APP expression between the two groups. This suggests that ACOT7 might have a significant involvement in amyloid pathogenesis of AD. However, the absence of ACOT7 does not appear to have an effect on the expression of metalloproteinase 10 (ADAM10), metalloproteinase 17 (ADAM17), and secreted APP alpha (sAPPα) (Figure 5D), possibly indicating ACOT7 may potentially play a role in regulating the metabolism of APP through the amyloidogenic pathway.

## 4 Discussion

In this study, a total of 366 cases from the AD group and 316 cases from the control group, matched by gender and age, were enrolled. Peripheral venous blood samples were collected from both groups to detect the serum levels of ACOT7. The identification of a more reliable biomarker in blood would hold significant significance and offer valuable assistance in the diagnosis of AD.

Previous reports pointed out that senile plaque formation was a typical and specific pathological feature of AD (O’brien and Wong, 2011). Aβ as the core structure of senile plaque, its abnormal deposition can produce the toxicity to nerve cells, affect learning and memory function, and is a key link in the pathogenesis of AD (Knowles et al., 2014; Paroni et al., 2019). The cleavage of APP produces many residues of 39 ~ 43 amino acids, of which Aβ_42_ and Aβ_40_ are the most common (Wilkins and Swerdlow, 2017). Aβ_42_ is hydrophobic, and is the most easily aggregated and deposited subtype with the strongest toxicity (Qiang et al., 2017). Aβ_42_, Aβ_40_ and P-Tau proteins in CSF have been included in the scientific research criteria for the diagnosis of AD (Charidimou et al., 2018; Mila-Aloma et al., 2020). Under normal circumstances, Aβ_42_, Aβ_40_ and P-tau proteins in peripheral blood and CSF can be in a balanced circulation state through the blood–brain barrier. Aβ_42_ was studied in CSF and blood in the large Swedish BioFINDER study cohort, and a significant correlation of Aβ_42_ was found between in plasma and in CSF (Hansson et al., 2018). MMSE score can be used to evaluate the degree of cognitive impairment in AD. Previous research has demonstrated a negative correlation between the expression level of Aβ_42_ and the MMSE score, indicating that Aβ_42_ in peripheral blood exhibits an increasing trend with cognitive decline (Giudici et al., 2020; Futamura et al., 2021). Our present study showed that ACOT7 were significantly increased in AD compared with the control group. Interestingly, there was a negative correlation between the serum ACOT7 protein level in AD group and MMSE scores (*r* = −0.85, *p* < 0.001), indicating ACOT7 might be related to Aβ production.

Using the siRNA technique, we achieved a significant reduction of ACOT7 expression in SK-N-SH APPwt cells by 88%, which could hold promise for future investigations. We investigated the effect of siACOT7 on APP metabolism, and found that siACOT7 could significantly reduce the amyloidogenic pathway of APP metabolism, while having no significant influence on the non-amyloidogenic pathway. The amyloidogenic processing of APP is carried out by the aberrant cleavage of β- and ϒ-secretases. β-secretase cleaves APP into CTFβ and sAPPβ. Therefore, the protein levels of CTEβ besides BACE1 and Aβ_42_ were also examined to confirm the subsequent effects after the reduction of BACE1. Our findings indicate that the downregulation of ACOT7 resulted in decreased levels of BACE1, Aβ_42_ and βCTF in SK-N-SH APPwt cells, as compared to the control group. The current focus on drug development targeting Aβ has emerged as a prominent area of research. However, many of these drug candidates have faced significant challenges, including severe side effects and unfavorable prognosis, leading to their discontinuation (Wojtunik-Kulesza et al., 2023; Yadollahikhales and Rojas, 2023; Zhang et al., 2023). In the present study, the involvement of ACOT7 in the production of Aβ through the amyloidogenic pathway of APP metabolism was demonstrated. This finding suggests that ACOT7 could potentially serve as a novel therapeutic target for the treatment of AD. Further investigation is needed to elucidate the exact roles of ACOT7 in the pathogenesis of AD.

The AD patients enrolled in this study did not distinguish between MCI and AD, and future studies should group them to investigate the differential expression of ACOT7 in Control, MCI and AD groups. At the same time, the earlier the diagnosis of AD, the better. Therefore, whether ACOT7 is highly expressed in the serum of MCI will also be the focus of subsequent research, and its application as a biomarker for early screening of AD will have greater significance. There were no deviations or other abnormalities that affected the reliability of the results and conclusions.

In summary, the protein expression of ACOT7 was demonstrated to be significantly increased in both AD patients and animal models of AD. The strong correlation observed between serum levels of ACOT7 and MMSE scores indicates a potential close association between ACOT7 and the severity of the disease. ROC analysis showed that the AUC of ACOT7 was 0.83, indicating that ACOT7 serves as a robust biomarker with high diagnostic accuracy in distinguishing AD patients from healthy individuals. The optimal cut-off point for ACOT7 was determined to be 62.5 pg./mL, resulting in a sensitivity of 80% and specificity of 74%. Accordingly, our results highlight potential of serum ACOT7 in identifying AD, thereby aiding in the diagnosis of AD.

## Data availability statement

The raw data supporting the conclusions of this article will be made available by the authors, without undue reservation.

## Ethics statement

The studies involving humans were approved by the ethics Committee on Human Experimentation of Ludong University. The studies were conducted in accordance with the local legislation and institutional requirements. The participants provided their written informed consent to participate in this study. The animal study was approved by the institutional guidelines of the Experimental Animal Center of Ludong University. The study was conducted in accordance with the local legislation and institutional requirements.

## Author contributions

JW: Data curation, Formal analysis, Investigation, Software, Supervision, Validation, Writing – original draft, Writing – review & editing. YF: Data curation, Funding acquisition, Investigation, Project administration, Resources, Validation, Writing – original draft, Writing – review & editing. YS: Funding acquisition, Investigation, Resources, Validation, Writing – original draft, Writing – review & editing.
